# The Early Recognition and Management of Sepsis in Sub-Saharan African Adults: A Systematic Review and Meta-Analysis

**DOI:** 10.3390/ijerph15092017

**Published:** 2018-09-15

**Authors:** Ben Morton, Marie Stolbrink, Wanjiku Kagima, Jamie Rylance, Kevin Mortimer

**Affiliations:** 1 Department of Clinical Sciences, Liverpool School of Tropical Medicine and Aintree University Hospital NHS Foundation Trust, Liverpool L9 7AL, UK; mstolbrink@doctors.org.uk (M.S.); kevin.mortimer@lstmed.ac.uk (K.M.); 2 Department of Clinical Sciences, Liverpool School of Tropical Medicine, Liverpool L3 5QA, UK; jacqueline.kagima@lstmed.ac.uk (W.K.); Jamie.Rylance@lstmed.ac.uk (J.R.); 3 Department of Medicine, Kenyatta National Hospital, Nairobi, P.O Box 20723-00202, Kenya; 4 Lung Health Group, Malawi-Liverpool-Wellcome Programme (MLW), Blantyre, P.O. Box 30096, Malawi

**Keywords:** sepsis, adults, sub-Saharan Africa, tuberculosis, pneumonia, protocolized care

## Abstract

Sepsis is a common cause of morbidity and mortality in sub-Saharan African adults. Standardised management pathways have been documented to improve the survival of adults with sepsis from high-resource settings. Our aim was to assess the current evidence base for early sepsis interventions (recognition, empirical antibiotics, and resuscitation) in resource-poor settings of sub-Saharan Africa. We searched MEDLINE, EMBASE and CINHAL Plus databases to identify interventional studies for the early recognition and management of sepsis in sub-Saharan Africa (1 January 2000 to 1 August 2018) using a protocol-driven search strategy: adults, protocolised care pathway, and sub-Saharan Africa. We identified 725 publications of which three met criteria for final selection. Meta-analysis from two randomised controlled trials demonstrated that mortality was increased by ‘early goal-directed therapy’ interventions that increased fluid resuscitation (R.R. 1.26, 95% C.I. 1.00–1.58, *p* = 0.045; *I*^2^ 53%). The third observational cohort study demonstrated improved survival after implementation of protocolised management for sepsis (mortality 33.0% vs. 45.7%, *p* = 0.005). No study incorporated standardised protocols for empirical antibiotic administration. High rates of pneumonia and mycobacteraemia were reported. There has been little research into the early recognition and management of sepsis in sub-Saharan Africa. Interventional trials of early goal-directed therapy have, to date, increased mortality. There is an urgent need to develop effective strategies to improve outcomes for adults with sepsis in sub-Saharan Africa.

## 1. Introduction

Sepsis—‘life threatening organ dysfunction caused by a dysregulated host response to infection’—affects approximately 30 million people around the world every year [1,2]. People in low- to middle-income (LMIC) areas of the world—and particularly sub-Saharan Africa—suffer disproportionately high mortality compared to those from high-income countries (HIC) [3]. The Surviving Sepsis Campaign (SSC Guidelines) [4] has promoted standardised early recognition and management of sepsis and led to improved patient outcomes in high-income countries [5]. The principles of early recognition, empirical antibiotics and fluid resuscitation set out in the SSC Guidelines have been adopted into sepsis management components of the World Health Organization (WHO) Integrated Management of Adolescent and Adult Illness (IMAI) guidelines in low-resource settings [6,7].

Whilst there are detailed Global Intensive Care working group of the European Society of Intensive Care Medicine and the World Federation of Paediatric Intensive and Critical Care Societies guidelines in place for adults admitted to critical care with sepsis in sub-Saharan Africa [8,9], a fundamental problem is that in the main, critical care facilities are lacking in the hospitals that these patients are admitted to [2,10,11,12,13,14]. Taking these realities into account, the WHO produced the IMAI guidelines that cover, including other illness presentations, the management of sepsis in low-resource settings [6,7]. Sepsis care bundles for children in sub-Saharan Africa have been extensively evaluated elsewhere [15].

The core components of early management for sepsis shared by the SSC and IMAI guidelines include: rapid identification, empirical antibiotics, resuscitation and adequate source control [4,6]. To date, there has been no systematic review that examines implementation of these principles in sub-Saharan Africa. Healthcare resource constraints including reduced provision of staff, drugs and equipment impact on the delivery of standardised care in this setting [2,9,11,13]. In addition, there are major demographic differences between patients with sepsis in higher-income compared to lower-income countries in sub-Saharan Africa including age, prevalence of HIV co-infection and ecology of pathogenic organisms [2]. In light of a recently published trial from Zambia that demonstrated worse outcomes when patients with sepsis were randomised to early goal-directed resuscitation [16], we set out to conduct a systematic review and meta-analysis of studies that examine early sepsis management in sub-Saharan Africa. We have focused on sub-Saharan Africa rather than a broader lower-middle income country review because of the specific characteristics of populations in this region. The majority of low-income countries are found in sub-Saharan Africa with associated severe healthcare resource constraints [17]. Furthermore, sub Saharan African populations suffer the highest prevalence of diseases such as HIV-infection [18] and tuberculosis (TB) [19]. Our aim was to determine the evidence base for key components of early sepsis management (early recognition, empirical antibiotics and fluid resuscitation) in the sub-Saharan African context.

## 2. Materials and Methods

### 2.1. Data Sources and Search Strategy

We searched the MEDLINE, EMBASE and CINAHL Plus databases for English language papers published between 1 January 2000 and 1 August 2018 using a systematic search strategy with the removal of duplicate titles. The bibliographies of studies indentified in the review were searched for any additional relevant titles. The review protocol was registered with PROSPERO (http://www.crd.york.ac.uk/PROSPERO, ID: CRD42017084606). Box 1 describes the search strategy; the full search strategy is in the supplemental material (Table S1).

Box 1 **Search terms for acute hospital presentation.**

Acute disease, sepsis, septic shock, bacteremia, HIV, HIV infections, AIDS-related opportunistic infections, tuberculosis, pulmonary tuberculosis, mycobacterium tuberculosis, pneumonia, bacterial pneumonia, pneumococcal pneumonia, straphylococcal pneumonia, mycoplasma pneumonia, viral pneumonia, virus diseases, mycoses, candidiasis, fungemia, hemorrhagic septicaemia, typhoid fever, paratyphoid fever.

The search terms for acute hospital presentation were combined with search terms for sub-Saharan Africa, adults and protocolised care pathways.

### 2.2. Study Selection

**Figure 1:**
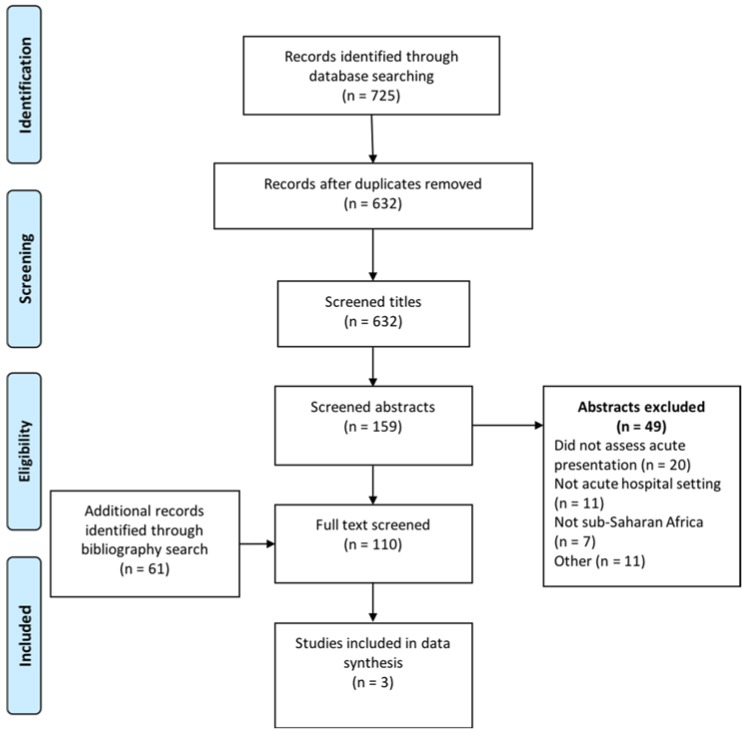
PRISMA flow diagram. PRISMA: Preferred Reporting Items for Systematic Reviews and Meta-Analyses.

Original observational studies and clinical trials that implemented treatment protocols for adults (≥18 years) admitted to hospital in sub-Saharan Africa with acute sepsis were included. The early recognition and management strategies were the interventions of interest and mortality was our primary outcome measure. Two authors (B.M. and M.S.) screened titles and abstracts and made study selection decisions independently. Papers that at least one author identified for inclusion were reviewed in full (Figure 1). There were no study design restrictions.

### 2.3. Data Extraction, Risk of Bias Assessment and Analysis

Two authors (B.M. and M.S.) extracted data on illness presentation, early recognition, physiological measurements, fluid therapy, antibiotic therapy, microbiological results, mortality and morbidity. Any disagreements between authors at the data extraction stage were arbitrated by a third independent author (J.R.). The Newcastle–Ottawa and Jadad scales were used to assess the methodological quality of observational studies and clinical trials, respectively [20,21]. Newcastle–Ottawa scores out of six and nine were used for cross-sectional and case-control/cohort studies, respectively. A narrative analysis of observational study data and a meta-analysis of clinical trial data (Review Manager (RevMan) [Computer program]. Version 5.3. The Nordic Cochrane Centre, The Cochrane Collaboration, Copenhagen, Denmark, 2014.) was completed.

## 3. Results

### 3.1. Study Selection

The initial search identified 725 studies of which 93 were duplicates, leaving 632 to be screened for eligibility. An additional 61 potentially eligible papers were identified through bibliography searching. After exclusion of 583 papers that did not meet the selection criteria, there were 110 papers left for full review. The most common reasons for excluding studies were: sepsis not examined, did not address acute hospital admissions, or were not conducted within sub-Saharan Africa. Three studies met the full selection criteria for data synthesis (Figure 1). In addition to the three studies included in the data synthesis (Table 1), we identified several key studies that addressed isolated components of sepsis management or were non-interventional in design. Although these studies fall outside of our selection criteria, they are summarised in Table 2 as they provide additional insights into sepsis management in sub-Saharan Africa.

### 3.2. Description of Included Studies

The three studies were published between 2012 and 2017 and included 1442 patients from two sub-Saharan African countries: Zambia [16,22] and Uganda [24]. Two were randomised controlled trials performed sequentially (see below) [16,22] and one was a prospective ‘before and after’ cohort study [24] (Table 1). The most common source of sepsis was pneumonia in all three studies: 103/209 (49.3%) [16], 63/109 (57.8%) [22] and the two randomised controlled trials [16,22] defined severe sepsis according to American College of Chest Physicians/Society of Critical Care Medicine 2001 guidelines (suspected infection, systemic inflammatory response syndrome and organ dysfunction [23]). However, the initial trial was stopped early after an interim analysis demonstrated that patients with severe respiratory distress were at potential risk of harm from the intervention (100% mortality vs. 70%, *p* = 0.09). In the follow-on trial, patients were excluded if they had hypoxaemia and tachypnoea at presentation [16]. Both studies identified delayed presentation to hospital in the study populations (median duration of symptoms for intervention and control groups 14 and 30 days [22] and median duration of inability to ambulate 16.5 and 10 days respectively [16]). In the cohort study done in Uganda, patients were selected based on a composite of suspected infection, physiological criteria and blood lactate levels (or Karnofsky performance scale) [23,24]. Time to presentation was not reported in this study.

### 3.3. Study Interventions

The intervention arm for the randomised controlled trials delivered a modified ‘early-goal directed therapy’ bundle of care within six hours of study enrolment to include early antibiotics, fluid, blood and vasopressor resuscitation; guided by physiological response. These interventions deviated from the original bundle described by Rivers et al. [31] due to logistical constraints such as lack of invasive monitoring (used alternative, more subjective measures) and critical care facilities. There was no significant difference in time to antibiotic administration for either study (median 1.5 vs. 1.3 h, *p* = 0.42 [22] and median 2.0 vs. 1.5 h, *p* = 0.15 [16]. However, neither study described a protocol for empirical antibiotic administration and both studies identified high incidences of mycobacteraemia in post hoc analyses. For example, *Mycobacterium tuberculosis* was identified in 43/209 (20.5%) patients recruited to the most recent trial [16] compared to 8/209 (3.8%) *Staphylococcus aureus*, 4/209 (1.9%) *Streptococcus pneumoniae*, 3/209 (1.4%) *Escherichia coli* and 3/209 (1.4%) *Klebsiella pneumoniae*. There were significant differences in volumes of fluid administered within six hours of enrolment in both studies (mean 2.9 L vs. 1.6 L, *p* < 0.001 [22] and median 3.5 L vs. 2.0 L, *p* < 0.001 [16]). The proportion of patients administered vasopressors (dopamine, 3/53 vs. 1/56 [22] and 15/106 vs. 2/103, *p* = 0.001 [16]) and blood (16/53 vs. 11/56, *p* = 0·20 [22] and 17/106 vs. 13/103, *p* = 0.48 [16]) was low in both studies.

The ‘before and after’ observational study used protocolised fluid resuscitation, guided by hourly monitoring, but did not standardise empirical antibiotic regimens [24]. Significantly more resuscitation fluid was given within 6 h of hospitalisation in the interventional compared to observational cohort (median 3.0 L vs. 0.5 L, *p* < 0.001). In addition, more patients received timely antibiotics (within 1 h of hospitalisation) in the interventional cohort (median 67.0% vs. 30.4%, *p* < 0.001); the majority of regimens were deemed inappropriate both intervention and observational cohorts (81.0% and 95.3%, respectively (*p* < 0.001)). The categorisation criteria for antibiotic appropriateness in this study were based on susceptibility of isolated organisms to the antibacterial agent prescribed or implementation of ‘normative’ local guidelines if no organism was isolated or susceptibility tests not performed.

### 3.4. Study Outcomes

**Figure 2:**
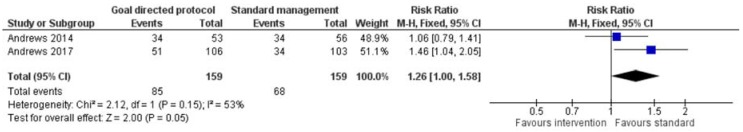
Forrest plot comparing mortality for a modified early goal directed therapy protocol versus standard management in patients with severe sepsis. Risk ratio less than one favours the intervention, more than one favours standard management. Heterogeneity: *χ*^2^ = 2.12, *p* = 0.145, *I*^2^ = 53%.

As shown in Figure 2, meta-analysis of data from the two randomised controlled trials found that mortality was increased by early goal directed therapy intervention (R.R. 1.26, 95% C.I. 1.00–1.58, *p* = 0.045; *I*^2^ 53%) [16,22]. In contrast, the observational cohort study [24] found mortality was decreased by early goal directed therapy intervention (adjusted H.R. 0.74, 95% C.I. 0.55–0.98). Both of the trials employed complex ‘bundles of care’ interventions such that it is difficult to distinguish individual factors impacting on outcome. A notable difference between the two trials and the observational cohort study was that patients in both the interventional and control arms of the randomised controlled trials received high volumes of resuscitative fluid (median 1.7–3.0 L) compared to the ‘before intervention’ cohort of the observational study (median 500 mL) [16,22,24]. The first randomised controlled trial (RCT) was stopped after interim analysis due to adverse mortality rates in patients with presentation hypoxaemic respiratory failure assigned to the interventional arm. In the second RCT, 61.3% (65/106) of patients in the intervention arm developed respiratory compromise that required cessation of fluid resuscitation [16].

### 3.5. Methodological Quality

For the three studies that met inclusion criteria, methodological quality was generally high (Table 1) although neither randomised controlled trial could be double-blinded and the cohort study employed separate ‘exposed’ and ‘non-exposed’ cohorts for comparison and did not control for important factors such as introduction of anti-retroviral therapy (ART) during the study period. The assessment criteria for the additional key studies is displayed in Table 1. Formal risk of publication bias assessment was not conducted due to the low number of studies detected for inclusion.

## 4. Discussion

This is the first systematic review and meta-analysis of early management strategies for adult patients with sepsis in sub-Saharan Africa. We found little existing research about pathways for the early recognition and treatment of sepsis in sub-Saharan African adults. Research to date has principally tested fluid resuscitation strategies with less attention to the appropriateness of empirical antimicrobial regimen for local infection ecology. Based on these studies, unselected liberal fluid resuscitation strategies were harmful for patients who present to hospital with sepsis in sub-Saharan Africa.

The majority of patients with sepsis do not have access to critical care therapies in sub-Saharan Africa [2,10,11,13]. Initial management is predicated on prompt recognition, appropriate early empirical antibiotic administration, organ support (e.g., fluid therapy and oxygen) and source control [4]. This bundle of care is a complex intervention developed and successfully implemented in patient cohorts from high-income countries to improve survival [32]. However, implementation of this approach for patients with sepsis in sub-Saharan Africa is at a preliminary stage with few evaluation studies that have addressed individual components of care.

Sepsis recognition in sub-Saharan Africa is a subject of controversy [33]: (1) updated sepsis definitions were based on patient cohorts from HICs with a low incidence of HIV-infection [1]. (2) Poor access to laboratory services in many settings effectively precludes the use of the ‘sequential organ failure assessment’ (SOFA) score recommended by these guidelines [1]. (3) The ‘quick’ SOFA tool [1], recently validated in LMIC settings (including sub Saharan African countries) to identify at-risk patients with sepsis [34], was not designed to discriminate patients with sepsis from patients without sepsis [26,35]. A ‘universal vital assessment’ (UVA) score has been proposed to identify acutely unwell adults in sub-Saharan Africa at risk of death (Table 1, [26]). However, implementation would likely be challenging due to critical shortages of both health care workers and material resources in sub-Saharan African healthcare settings [36] and this tool has not yet been used to direct patient care in clinical trials. For example, a retrospective cohort study in Uganda demonstrated that the median monitoring frequency was 1.1/day for blood pressure and 0.5/day for respiratory rate in patients admitted to medical wards [37]. For an effective rapid response system, the afferent recognition limb must trigger an efferent ‘action’ limb to manage the at-risk patients [38]. Based on HIC evidence, this issue is especially important for patients with sepsis as early administration of appropriate antibiotics promotes significantly reduced mortality [23]. In future, converging technological advances such as open source software, cheaper hardware, and improved information technology could promote translation of electronic medical records for hospital in-patients in sub-Saharan Africa [39]. Smartphone health informatics may be particularly useful in this context [40]: direct recording of physiological parameters onto electronic handheld devices (potentially by low-skilled staff) could automatically direct medical attention to the ‘at-risk’ patient.

Early administration of appropriate empirical antibiotics for bacterial sepsis is strongly associated with reduced mortality in high-income settings [32,41]. A large prospective cohort study (*n* = 49,331) conducted in the US found that the odds ratio for death was 1.04 (CI: 1.03–1.06) for every hour in delayed administration [32]. For LMICs, WHO IMAI guidelines recommend that administration of empirical antibiotics within the first hour of sepsis recognition is ‘crucially important’ [6]. The antibiotics recommended for bacterial sepsis in LMICs are intravenous ceftriaxone (1 g daily) or ampicillin (2 g four hourly) with gentamicin (1.5 mg/kg eight hourly) [6]. These combinations empirically cover antimicrobial sensitive Gram-positive and Gram-negative pathogens but not TB, common in sub-Saharan Africa due to the high prevalence of co-existing HIV-infection [42]. TB, invasive mycoses such as Cryptococcal meningitis and malaria are well recognised causes of sepsis in this context [43,44,45]. There are, however, no studies that have investigated the effect of standardised early antimicrobial therapy for these infections on patient outcome.

A ‘step-up’ approach to antimicrobial administration is commonly employed for sepsis in sub-Saharan Africa; this may limit expensive and complex treatment but could promote harmful treatment delays [46]. This non-empirical, pathogen-specific approach to management, combined with health service constraints and limited adherence to antimicrobial guidelines [29] has restricted antibiotic treatment effectiveness to date [47]. Currently, there are limited context-specific microbiological data to guide appropriate selection of empirical antimicrobials in sub-Saharan Africa [7]. A systematic review of community-acquired bacteraemia in Africa found that *Salmonella enterica* (majority non-typhoidal, 29.1%), *Streptococcus pneumoniae* (18.3%)*, Staphylococcus aureus* (9.5%) and *Escherichia coli* (7.3%) were the most common bacterial isolates [25]. Laboratory services in sub-Saharan Africa are frequently lacking [25]; limited by cost, infrastructure and personnel constraints [48]. A major issue, not adequately addressed by current guidelines is the high prevalence of disseminated TB in patients with HIV-infection and sepsis [16,22,48]. When appropriate TB blood culture techniques are used, mycobacteraemia can be detected in up to 30.7% of samples [25]. Such infections typically present with non-specific clinical signs [49,50], sputum culture has poor sensitivity with acutely unwell patients frequently unable to produce a sample [51], and chest x-rays non-discriminatory for diagnosis [28,52]. The intervention studies included in this review did not use protocols for empirical antibiotics [16,22,24], and this raises the possibility that the antimicrobials used in these studies did not provide sufficient empirical cover.

The advent of microbiological point-of-care testing such as urine lipoarabinomannan (LAM, a glycolipid antigen from the mycobacterial cell wall) may be of particular value for sepsis in sub-Saharan Africa. Recently, a randomised controlled trial demonstrated that urinary LAM testing combined with therapeutic recommendations reduced time to initiation of anti-TB therapy compared to standard investigation alone (55% commenced day one vs. 40%, *p* < 0.0001) and eight-week mortality (21% vs. 25%, *p* = 0.012) for patients admitted to hospital with suspected TB and HIV co-infection [28]. Furthermore, a pragmatic randomised controlled trial that assessed the use of urinary LAM testing for all patients with HIV-infection admitted to medical wards (STAMP [27], intervention did not include a specific treatment recommendation) demonstrated earlier TB diagnosis and improved mortality for particularly at-risk patient subgroups (CD4 < 100, severe anaemia and clinically suspected TB). In future, a structured urinary LAM test and anti-tuberculous therapy intervention to further reduce time to therapy could potentially be trialed to determine if outcomes for patients with sepsis can be improved.

Emerging research from higher-income countries questions the benefits of aggressive fluid resuscitation for patients with sepsis [32]. In a large prospective cohort study (New York State, U.S., *n* = 49,331), investigators found that early delivery of resuscitative fluids did not improve outcome (O.R. mortality 1.01 per hour delay, C.I. 0.99–1.02, *p* = 0.21) [32]. Based on published randomised controlled trial evidence in sub-Saharan Africa to date, liberal fluid strategies guided by clinical measures of physiological response are likely to cause harm [16,22]. It should be noted that patient recruitment in the first trial [22] was stopped early due to safety concerns and that much more work is required in this area before readers can draw definitive conclusions. Results from the observational study suggest that minimal fluid resuscitation for sepsis may also be harmful [24]. However, this ‘before and after’ study was conducted between November 2006 and May 2009 during which time confounding factors such as ART roll-out may have impacted on results. For septic patients in sub-Saharan Africa, where fluid resuscitation is often the sole therapy to correct septic shock, a key research issue is to define pragmatic cut-off points for safe volumes of fluid resuscitation. We would add a caveat that these findings are not applicable for specific infectious disease outbreaks that cause severe diarrhoea and vomiting (e.g., cholera and the recent Ebola outbreaks) where fluid resuscitation to prevent hypovolaemic shock is essential [53,54].

Adequate source control is an essential component of sepsis management; IMAI guidelines suggest drainage of any surgical infection within 2–6 h of sepsis diagnosis [6]. However, obstetric and surgical management for septic conditions is frequently delayed in sub-Saharan Africa [55]. As a result, damage control surgery must frequently be employed with multiple subsequent returns to theatre, burdensome in a low-resource health care setting [55]. Where surgical delay is unavoidable, percutaneous drainage of uncontrolled abdominal infection or hydronephrosis have been advocated [55]. Potentially, point-of-care ultrasound could also be used to rapidly identify and treat other drainable collections (e.g., empyema) [56].

As an additional point, the provision of oxygen has been described as an ‘entry point’ for improving quality of care in lower-middle income countries [57] and is a vital component of sepsis management [4]. However, availability is extremely limited in sub-Saharan Africa [11,13,58,59]: one study identified that only 44% of surveyed facilities (12 countries) had an uninterrupted supply of oxygen [30]. Another issue is that directed delivery of oxygen is dependent on the measurement of hypoxaemia (i.e., by pulse oximetry). Recently, interventions such as the Global Pulse Oximetry Project [59] are improving access to this vital equipment [26]. Currently, an implementation trial is underway in Papua New Guinea to assess the effectiveness of solar-powered oxygen concentrator systems in remote health care centres [57]. The results of this trial will potentially be useful to guide wider implementation of oxygen therapy in sub-Saharan Africa.

This is the first systematic review of early management strategies for patients with sepsis in sub-Saharan Africa. The strengths of this review include: a broad search strategy to identify relevant evidence for the implementation of sepsis care bundles in sub-Saharan Africa and rigorous implementation of PRISMA guidelines. Few interventional studies have been published in this area; this limits our ability to conduct a quantitative synthesis and measure potential publications bias. Furthermore, we have not been able to disaggregate data from care bundles used in these interventional studies to define potential benefits of individual treatment components. An additional limitation is that we restricted studies to the English language only.

## 5. Conclusions

We found little evidence concerning the early recognition and management of sepsis in sub-Saharan Africa. To date, published interventional studies have applied complex bundles of care developed in higher-income countries to sub-Saharan Africa and not shown benefit. Further research is required to examine how tools such as the ‘universal vital assessment’ score can be applied to track at-risk patients and trigger effective management strategies, potentially using innovative and pragmatic information technologies. In the presence of multiple potential pathogens, non-specific sepsis presentations and limited laboratory services, standardised application of rapid point-of-care tests to identify a combination of, for example, HIV-infection, malaria, TB and pneumococcal disease could be used to tailor structured empirical antimicrobial approaches in the future. Based on current evidence, recommendations for aggressive fluid resuscitation should be revised in the sub-Saharan African setting and research is urgently needed to identify pragmatic ‘stop-points’ to guide personalised resuscitation. Context-sensitive piloting of individual care bundle components is required to determine efficacy and feasibility in the sub-Saharan setting.

## Figures and Tables

**Table 1:** Interventional studies that describe early goal-directed therapy for sepsis in sub-Saharan Africa.

Study	Design	Bias	Country	Participants	Population	Age	Crude Mortality
Andrews, 2014 [22]	Randomised controlled trial (RCT)	4/5	Zambia	112	Accident and emergency (A & E) with severe sepsis [23]	Intervention (35.2, 1.3) ^α^ Control (34.8, 1.4) ^α^	Intervention: 64.2% (*n* = 53) Control: 60.7% (*n* = 56)
Andrews, 2017 [16]	RCT	4/5	Zambia	212	A & E with severe sepsis [23]	Intervention (37.5, 12.9) ^α^ Control (35.8, 11.9) ^α^	Intervention: 48.1% (*n* = 106) Control: 33.0% (*n* = 103)
Jacob, 2012 [24]	Prospective Cohort	7/9	Uganda	671	Medical ward with sepsis (study specific criteria)	Intervention (34, 27–40) ^β^ Observation (34, 28–41) ^β^	Intervention: 33.0% (*n* = 426) Observation: 45.7% (*n* = 245)

Risk of bias assessments: Newcastle–Ottowa scale and Jadad scale for cohort studies and randomised controlled trials, respectively [20,21]. Age: *α* = mean and standard deviation (SD); *β* = median and interquartile range (IQR).

**Table 2:** Additional studies that did not meet full inclusion criteria but examined components of early sepsis management included in the review.

Study	Design	Bias	Country	Participants	Population	Study Summary
Reddy, 2010 [25]	Systematic review		African continent	58,296 (adults and children)	Patients with ≥1 blood culture	Reports bacterial pathogens isolated from blood cultures: *S. enterica*, *S. pneumoniae*, *S. aureus* and *E. Coli.* Mycobacterium tuberculosis frequently isolated (166/539) where appropriate techniques used.
Moore, 2017 [26]	Systematic review		Sub-Saharan Africa	5573	Adults admitted to hospital	Pooled data from 13 cohort studies to derive a ‘universal vital assessment’ score to predict in-hospital mortality based on physiological parameters. Clinical variables include: temperature, heart rate, respiratory rate, systolic blood pressure (BP), SpO_2_, Glasgow coma scale (GCS) and HIV-infection status. Score AUCROC for mortality 0.77 (0.75–0.79).
Gupta-Wright, 2018 [27]	RCT	5/5	Malawi and South Africa	4788	Adult inpatients with HIV-infection	Urinary lipoarabinomannan guided therapy did not reduce overall mortality (adjusted risk reduction [aRD]—2.8%, CI—5.8 to 0.3; *p* = 0.074) but did reduce mortality in pre-defined subgroups with CD4 count <100, severe anaemia and clinically suspected tuberculosis (TB).
Peter, 2016 [28]	RCT	4/5	Sub-Saharan Africa	2659	Adult admissions with HIV-infection and TB symptoms	Urinary lipoarabinomannan guided anti-tuberculosis treatment reduced initiation time (median 0 day [IQR 0–2] vs. 1 day [IQR 0–3), *p* < 0.0001) and eight-week mortality (21% vs. 25%, ARR 4% C.I. 1–7%).
Jacob, 2009 [29]	Prospective Observational	6/9	Uganda	382	A & E with severe sepsis [23]	Management and outcomes of patients with severe sepsis: including poor fluid resuscitation (median 500 mL within 6 h) and antibiotic administration (61% patients received within 6 h) following sepsis diagnosis. Thirty-day mortality 43.0%.
Belle, 2010 [30]	Cross-sectional	6/9	Multiple (African continent)	231 hospitals and health centres	Healthcare facilities	Snapshot survey assessed oxygen supply and infrastructure in 12 African countries. Only 43·8% of facilities had uninterrupted access to an oxygen source and 24·6% had a fully functioning oxygen concentrator. Electricity fully available at 35·1% of facilities

Risk of bias assessments: Newcastle–Ottowa scale and Jadad scale for cohort studies and randomised controlled trials, respectively. AUCROC: Area Under Receiver Operator Curve; ARR: Absolute risk reduction.

## Supplementary Materials

The following are available online at http://www.mdpi.com/1660-4601/15/9/2017/s1, Table S1: Search Strategies.
