# Supplementary material for: The Early Recognition and Management of Sepsis in Sub-Saharan African Adults: A Systematic Review and Meta-Analysis
Source: Int J Environ Res Public Health. 2018 Sep 15;15(9):2017. doi: 10.3390/ijerph15092017 (PMC6164025; doi:10.3390/ijerph15092017)
Supplement: Supplementary file 1 [file ijerph-15-02017-s001.pdf]

International Journal of
Environmental Research and Public Health

MDPI

Table S1. Search Strategies.

PubMed	1) Adult OR Aged OR Middle Aged OR Young Adult<br>2) Developing countries OR AFRICA, WESTERN/ or AFRICA, CENTRAL/ or "AFRICA SOUTH OF THE SAHARA"/ or AFRICA/ or SOUTH AFRICA/ or AFRICA, EASTERN/ or AFRICA, SOUTHERN/<br>3) Acute disease OR Shock, septic OR Sepsis OR Bacteremia OR Virus Diseases OR Mycoses OR Candidiasis/ or Fungemia/ or Candidemia/ OR HIV OR Endotoxaemia OR Hemorrhagic Septicemia OR HIV infections OR AIDS-related opportunistic infections OR Tuberculosis OR Mycobacterium Tuberculosis OR PNEUMONIA, PNEUMOCOCCAL/ or PNEUMONIA/ or PNEUMONIA, MYCOPLASMA/ or PNEUMONIA, STAPHYLOCOCCAL/ or PNEUMONIA, NECROTIZING/ or PNEUMONIA, ASPIRATION/ or PNEUMONIA, VIRAL/ or PNEUMONIA, BACTERIAL/ OR Typhoid Fever OR Paratyphoid Fever<br>4) Triage OR “Severity of illness index” OR resource allocation OR health care rationing OR Decision making OR Health priorities OR Patient Selection<br>1 AND 2 AND 3 AND 4
Embase	1) exp AGED OR exp Middle Aged OR exp Young Adult OR exp Adult<br>2) exp Developing Countries OR exp Africa, Western/ OR exp Africa, Central/ OR exp Africa, South of the Sahara/ OR exp Africa, Nothern/ OR exp South Africa/ OR exp Africa, Eastern/ OR exp Africa. Southern/<br>3) exp Acute disease/ OR exp Shock, septic OR exp Sepsis OR exp Bacteremia OR exp Virus Diseases OR exp Mycoses OR exp Candidiasis/ OR exp Fungemia OR exp HIV OR exp hemorrhagic septicaemia OR exp AIDS-related Opportunistic Infections OR exp Tuberculosis OR exp Tuberculosis, pulmonary OR exp Mycobacterium Tuberculosis OR exp HIV Infections OR exp Pneumonia OR exp Pneumonia, Bacterial OR exp Pneumonia, pneumococcal OR exp Pneumonia, Staphylococcal OR exp Pneumonia, Mycoplasma OR exp Pneumonia, Viral OR exp Typhoid Fever OR exp Paratyphoid<br>4) exp Triage OR exp “Severity of Illness Index” OR exp Resource Allocation OR exp Health Care Rationing OR exp Decision OR exp Health Priorities OR exp Patient Selection<br>1 AND 2 AND 3 AND 4
CINHAL PLUS	1) Adults OR aged OR middle aged OR young adults<br>2) Developing countries OR Africa<br>3) Acute disease OR septic shock OR sepsis OR septic OR severe sepsis OR SIRS OR systemic infection OR bacteraemia OR blood stream infection OR virus disease OR mycoses OR candidiasis OR fungemia OR candidemia OR HIV OR Endotoxemia OR hemorrhagic septicaemia OR AIDs related opportunistic infections OR tuberculosis OR pulmonary tuberculosis OR mycobacterium tuberculosis OR HIV infection OR pneumonia OR bacterial pneumonia OR pneumococcal pneumonia OR staphylococcal pneumonia OR mycoplasma pneumonia OR viral pneumonia OR typhoid fever OR paratyphoid fever<br>4) Triage OR “Severity of illness index” OR resource allocation OR healthcare rationing OR decision making OR health priorities OR patient selection<br>1 AND 2 AND 3 AND 4
